# Ecological Momentary Assessment Using Smartphones in Patients With Depression: Feasibility Study

**DOI:** 10.2196/14179

**Published:** 2021-02-24

**Authors:** Redwan Maatoug, Nathan Peiffer-Smadja, Guillaume Delval, Térence Brochu, Benjamin Pitrat, Bruno Millet

**Affiliations:** 1 Sorbonne Université, AP-HP Service de psychiatrie adulte de la Pitié-Salpêtrière Institut du Cerveau, ICM, F-75013 Paris France; 2 National Institute for Health Research Health Protection Research Unit in Healthcare Associated Infections and Antimicrobial Resistance Imperial College London London United Kingdom; 3 French Institute for Medical Research (Inserm), Infection Antimicrobials Modelling Evolution UMR 1137 University Paris Diderot Paris France

**Keywords:** ecological momentary assessment, depression, smartphone, feasibility study, user experience

## Abstract

**Background:**

Ecological momentary assessment (EMA) is a promising tool in the management of psychiatric disorders and particularly depression. It allows for a real-time evaluation of symptoms and an earlier detection of relapse or treatment efficacy. The generalization of the smartphone in the modern world offers a new, large-scale support for EMA.

**Objective:**

The main objective of this study was twofold: (1) to assess patients’ compliance with an EMA smartphone app defined by the number of EMAs completed, and (2) to estimate the external validity of the EMA using a correlation between self-esteem/guilt/mood variables and Hamilton Depression Rating Scale (HDRS) score.

**Methods:**

Eleven patients at the Pitié-Salpêtrière Hospital, Paris, France, were monitored for 28 days by means of a smartphone app. Every patient enrolled in the study had two types of assessment: (1) three outpatient consultations with a psychiatrist at three different time points (days 1, 15, and 28), and (2) real-time data collection using an EMA smartphone app with a single, fixed notification per day at 3 pm for 28 days. The results of the real-time data collected were reviewed during the three outpatient consultations by a psychiatrist using a dashboard that aggregated all of the patients’ data into a user-friendly format.

**Results:**

Of the 11 patients in the study, 6 patients attended the 3 outpatient consultations with the psychiatrist and completed the HDRS at each consultation. We found a positive correlation between the HDRS score and the variables of self-esteem, guilt, and mood (Spearman correlation coefficient 0.57). Seven patients completed the daily EMAs for 28 days or longer, with an average response rate to the EMAs of 62.5% (175/280). Furthermore, we observed a positive correlation between the number of responses to EMAs and the duration of follow-up (Spearman correlation coefficient 0.63).

**Conclusions:**

This preliminary study with a prolonged follow-up demonstrates significant patient compliance with the smartphone app. In addition, the self-assessments performed by patients seemed faithful to the standardized measurements performed by the psychiatrist. The results also suggest that for some patients it is more convenient to use the smartphone app than to attend outpatient consultations.

## Introduction

A promising development in the treatment of unipolar depression consists of improving the monitoring of depressive symptoms at home. Indeed, a personalized follow-up and continuous assessment of the symptomatology and its contextual inﬂuences are paramount to the management of depression.

Ecological momentary assessment (EMA) is a method used in psychiatric research that collects real-time data on symptoms, microenvironmental fluctuations, and medication intake in the everyday environment of patients.

Several reviews have confirmed an interest in EMA in various psychiatric disorders [1-3], particularly mood disorders [4-6] such as depression [7,8]. In the context of mood disorders, symptoms are subjective and usually fluctuate from day to day. Consequently, the evaluation by a clinician at each consultation is necessary but is less precise than a continuous self-assessment. Furthermore, studies such as the one by Ben-Zeev et al [9] highlighted that during consultation patients tended to talk only about the negative aspects of their recent history and forgot about the positive ones. EMA helps reduce this bias and improves our understanding of mood fluctuations, including their links with the environment and medication adherence [10,11].

The first EMA studies were published in the 1980s by Csikszentmihalyi and LeFevre [12]. The authors were interested in knowing whether the quality of human experience was more influenced by whether a person was at work or at leisure or by whether a person was in “flow” (ie, a condition of optimal experience created when one’s environment presents high challenges that are met by one’s skills). The first EMA studies in depression, conducted using paper-and-pencil daily diaries, were published in the 1990s [13].

In previous EMA studies, data were mostly collected using a notebook that the patient had to complete. To avoid missing data, patients were reminded by a signal to write in the notebook. This method suffered from the risk of recall bias, as it was never certain when the patient filled in the questionnaire. In that regard, Stone et al [14] showed that “although patients reported high compliance, actual compliance was low.” Using the patient’s smartphone makes it possible to record the time of data collection. Over the last 10 years, smartphone use has become widespread in the population. While the penetration rate of smartphones was 29% in the French population in 2012, it exceeded 65% in 2018, offering an interesting tool for real-time monitoring of symptoms [15,16]. However, EMA studies using a smartphone app associated with an online platform are still scarce [17,18], although a few studies have used EMA apps on the smartphones of participants with depressive symptoms and/or bipolar disorder [19].

Recent EMA studies that included data restitution to patients have demonstrated that patients’ knowledge of their mood fluctuations and their context can assist them in understanding and managing their pathologies and, consequently, allow them to switch from passive consumers into active participants in their own care [20]. This innovative use of EMA may have the potential to improve prediction of relapse and remission [21,22].

Finally, a few studies have been published exploring the benefits of using a dashboard to provide data restitution and feedback from the clinician to the patient using the EMA data collected. Simons et al [20,23] demonstrated the therapeutic interest in restoring patient results from the EMA in a randomized trial involving approximately 102 patients with depression. They explored not only the collection of clinical information and their predictive values in terms of prognostic evaluations but also the return of this information to patients living with depression and the economic consequences of such a method.

Thus, coupling EMA with the patient’s smartphone has three main goals: (1) the possibility of real-time and continuous evaluation of symptoms [24], (2) early detection of relapse and treatment efficiency [25], and (3) generalization of the tool on a large scale [26,27].

The aim of our work was to highlight the relevance of using EMA with smartphones to track patients with depression. The main objective of this study was twofold: (1) to assess patients’ compliance with an EMA smartphone app defined by the number of EMAs completed, and (2) to estimate the external validity of the EMA using a correlation between self-esteem/guilt/mood variables and Hamilton Depression Rating Scale (HDRS) score.

## Methods

This 28-day, single-center prospective study took place between August and October 2019. During the study, patients had continuous full access to the EMA app on their smartphone.

### Population

All patients included in the study were experiencing a major depressive episode according to the Diagnostic and Statistical Manual of Mental Disorders, Fifth Edition criteria, with a recent introduction (less than one month) or modification of an antidepressant. Additional inclusion criteria were ownership of a smartphone and age over 18 years. Patients were included over a 2-week period.

The study took place at the psychiatry department of the Pitié-Salpêtrière Hospital (Paris, France). With the help of the psychiatrist, the patients downloaded the EMA app onto their personal smartphone. Additionally, they were informed that the clinical data were processed in real time and reviewed during the outpatient consultations with the psychiatrist.

### Assessments

Every patient enrolled in the study was expected to have two forms of assessment: (1) three outpatient consultations with a psychiatrist at three different time points (days 1, 15, and 28), and (2) real-time data collection using the EMA smartphone app, with a single, fixed notification daily at 3 pm.

#### Outpatient Consultations

During the three outpatient consultations, the psychiatrist assessed anxiety and depressive symptoms using the HDRS. The HDRS is a 17-item questionnaire designed for adults and used to rate the severity of their depression from 0 (no symptoms of depression) to 52 (severe depression) by probing mood, feelings of guilt, suicide ideation, insomnia, agitation or retardation, anxiety, weight loss, and somatic symptoms [28].

#### Smartphone App

**Figure 1:**
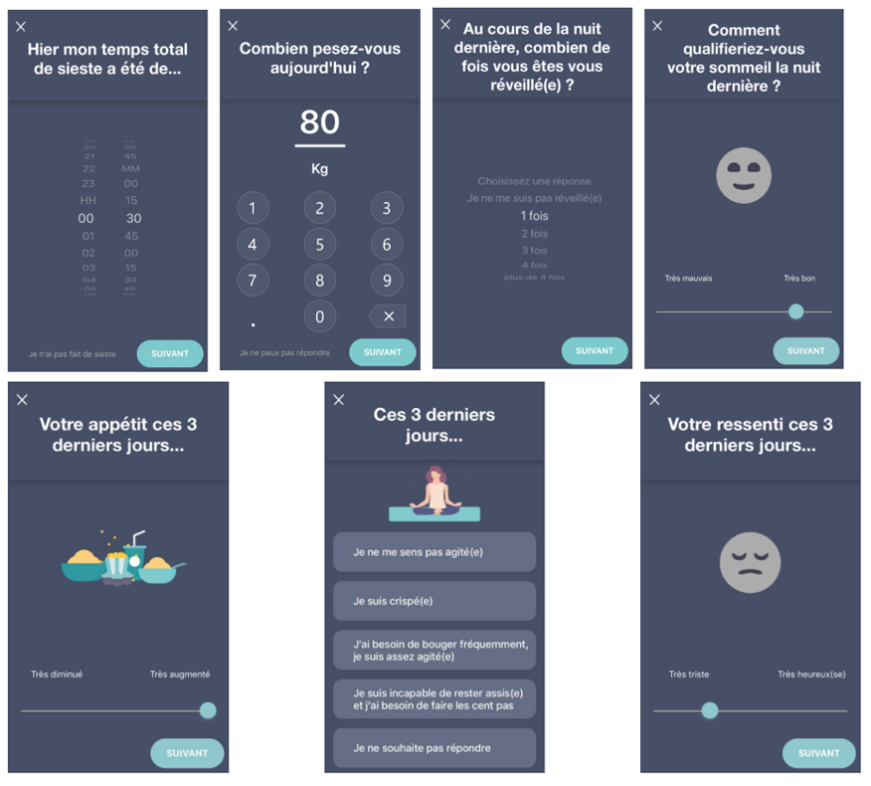
Illustration of the ecological momentary assessment smartphone app.

The EMA smartphone app is available in both iPhone (iOS) and Android operating systems (Figure 1).

Every day at 3 pm, each patient received a notification on their smartphone to remind them to respond to the EMA questions. However, patients could choose which questions they wished to answer. Indeed, a drop-down menu is present on the app and allows the patient to choose between different questions. This tool was developed to allow the patient to answer any particular question according to their mood or preference. Therefore, patients were not obliged to answer all of the questions and could stop at any time.

Each patient was asked to answer 10 questions per day for a period of 28 days, for a total of 280 EMAs to be completed over the course of the study. In order to assess the compliance of patients to the smartphone app in their home environment, no external reminders were sent to the patients.

The data collected with the smartphone app were as follows: antidepressant compliance (binary answer); potential side effects of the antidepressant (binary answer); mood evaluation (“Could you please rate your mood over the last 3 days?”); self-esteem (“How self-confident have you been during the last 3 days?”); guilt (“How guilty did you feel during the last 3 days?”); sleep parameters (bedtime and time to fall asleep, sleep quality); appetite (binary answer and weight changes); and general interest.

Each visual analog scale used semantic differentials as anchor points for the patient, which were converted to 0 to 100 for the clinician.

### Study Design

The study was developed in collaboration with Ad Scientiam, a French start-up specialized in real-life data, an initiative of the Brain and Spine Institute at the Pitié Salpêtrière Hospital in Paris, France.

No direct nominative data were collected in this study. All participants were identified by an identification code in the app. The study was approved by the local ethics committee for the protection of persons at Sorbonne University and by the Chair of the data protection commission (Commission nationale de l’informatique et des libertés). Written informed consent was obtained from all participants in the study.

## Results

### General Description

Eleven patients experiencing a major depressive episode were included in this feasibility study. All patients downloaded the EMA app onto their personal smartphone (Table 1).

**Table 1:** Summary of compliance with the smartphone app and outpatient consultations for each patient.

Patient	Gender	Age (years)	Days of follow-up^a^	Number of EMAs^b^ completed^c^	Number of outpatient consultations attended^d^
1	Female	45	35	300	2
2	Male	52	35	252	3
3	Male	51	35	106	3
4	Female	41	33	280	3
5	Female	37	30	297	2
6	Male	45	29	210	3
7	Male	56	28	56	3
8	Female	57	17	92	3
9	Male	70	11	53	1
10	Male	67	8	78	1
11	Male	69	5	32	1

^a^The duration of follow-up was 28 days. Some patients exceeded 28 days because they continued to use the app after the end of the study.

^b^EMAs: ecological momentary assessments.

^c^The number of EMAs completed during the total follow-up period; data collected after the 28th day were included in the total number, as the objective was to measure patient compliance with the smartphone app.

^d^Three outpatient consultations were scheduled (on days 1, 15, and 28).

**Figure 2:**
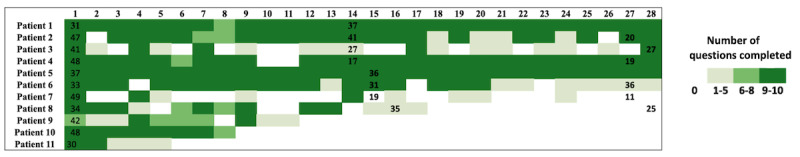
Monitoring of each patient for the 28 days of the study. The first value for each patient corresponds to the Hamilton Depression Rating Scale (HDRS) score at the first outpatient consultation (day 1); the second and third values correspond to the HDRS scores at the second (day 15) and third (day 28) outpatient consultations, respectively. The absence of a value indicates that the patient missed the consultation. The intensity of the green bar correlates with the number of questions out of a total of 10 that were answered in a given day.

Of the 11 participants, 6 patients attended the three outpatient consultations with the psychiatrist and completed the HDRS at each consultation. Seven of the 11 patients responded to the EMAs for a duration of 28 days or longer; the mean duration of follow-up was 24 days. It should be noted that of the 7 patients who completed the EMAs for a minimum of 28 days, 6 patients continued to complete them after the end of the study; patients 1 and 5 continued to complete EMAs without attending outpatient consultations with the psychiatrist (Figure 2).

### EMAs and HDRS Score Correlation

On average, 175 (175/280, 62.5%) EMAs were completed by each patient. The least compliant patient in the smartphone app responded to 53 EMAs and the most compliant patient responded to 278 EMAs.

When we focused on the six patients who completed the HDRS three times, we found a positive correlation between the average HDRS score and the average score for the variables of self-esteem, guilt, and mood (Spearman correlation coefficient 0.57).

Furthermore, there was a correlation between the number of responses to EMAs and the duration of follow-up. In fact, the higher the response rate to EMAs, the longer the follow-up (Spearman correlation coefficient 0.63).

## Discussion

This is the first feasibility study of an EMA tool with daily notifications for 28 days in patients experiencing a major depressive episode.

Our first objective was to assess the compliance to the smartphone app using the number of EMAs answered. Of the 11 patients, 7 patients responded to the EMAs for a duration of 28 days or longer; the mean duration of follow-up was 24 days. Moreover, on average, 175 responses (out of 280 possible responses; 62.5%) to EMAs were given. Surprisingly, we observed that two patients preferred answering the EMAs rather than attending the outpatient consultations with the psychiatrist.

**Figure 3:**
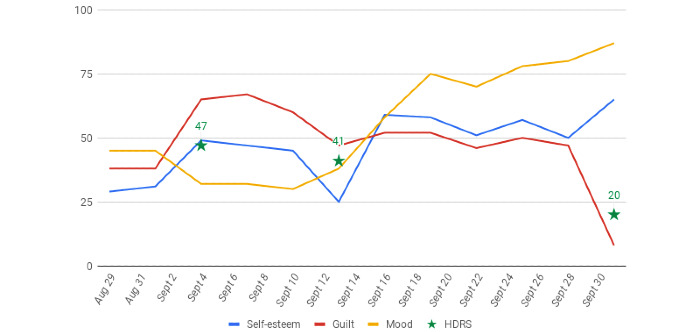
Dashboard used by the psychiatrist to visualize the patient’s data during the outpatient consultation. The psychiatrist chose which variable to display (eg, antidepressant compliance, mood evaluation), on which statistical graph (eg, pie chart, histogram, box plot), and over which period of time (eg, daily, weekly, monthly). Aug: August; HDRS: Hamilton Depression Rating Scale; Sept: September.

The second objective was to estimate the external validity of the EMA using a correlation between self-esteem/guilt/mood variables and HDRS score (Figure 3). Interestingly, patients seemed to assess their self-esteem, guilt, and mood as accurately as a psychiatrist using the standardized HDRS (Spearman correlation coefficient 0.57); these results are consistent with the literature, particularly the papers by Faurholt-Jepsen et al [29,30] and Dogan et al [4]. According to Cuijpers et al [31], self-report measures and clinical assessment are not equivalent but may provide complementary information.

By comparison, a feasibility study by Husky et al [32], which was conducted over 3 consecutive days with a preprogrammed personal digital assistant, showed an average compliance of 82% in the group of patients with major depression and 86.7% in the group of patients with any mood disorder. Johnson et al [33] found a 78% compliance rate after 1 week of follow-up in patients with severe psychiatric disorders.

Although a few studies, such as one by Vachon et al [34], have reported a high completion rate in patients with depression after 5 months of follow-up, the lower completion rate in our study may have been a result of the long duration of follow-up. In fact, in our study, patients had to complete the EMAs for 28 days, while in the studies by Husky et al [32] and Johnson et al [33], the follow-up periods were 3 days and 1 week, respectively. In addition, fixed notifications at precise times may be less disruptive than random notifications if they appear at a convenient time to the respondent.

Our results are encouraging, as they tend to confirm our initial hypothesis that data collected by patients using their own smartphone are more reliable than data obtained by a clinician during standard medical follow-up. Additionally, real-time data collection highlights the variability in the symptomatology of depression during the introduction or modification of an antidepressant. The analysis of patterns of emotional variability might lead practitioners to improve their understanding of mood disorders and might help us to identify risk factors for depression relapse.

At the patient level, the results obtained for the various self-assessment criteria—in this case mood, self-esteem, guilt, and sadness—allowed us to obtain, thanks to the large volume of data collected, a reliable representation of the symptoms expressed by the patient during the entire study. The availability of such a user-friendly tool could actually lead to a greater investment by the patient in his/her own care.

Our study also points out the difficulties for some patients in using smartphones. In our data, we observed many inconsistent results, especially for older patients, suggesting that they could not master the slide function on their device. In the future, more emphasis should be placed on assisting patients in the use of smartphones.

## Abbreviations

EMA: ecological momentary assessment
HDRS: Hamilton Depression Rating Scale
